# Transforming the Water‐Energy Nexus in Gaza: A Systems Approach

**DOI:** 10.1002/gch2.202300304

**Published:** 2024-03-12

**Authors:** Tony Rantissi, Vitaly Gitis, Zhiyuan Zong, Nick Hankins

**Affiliations:** ^1^ Faculty of Engineering Sciences Ben‐Gurion University of the Negev PO Box 653 Beer‐Sheva 8410501 Israel; ^2^ Laboratory of Sustainable Water Engineering Department of Engineering Science University of Oxford Parks Road Oxford OX3 1PJ UK; ^3^ Lady Margaret Hall University of Oxford Norham Gardens Oxford OX2 6QA UK

**Keywords:** energy planning, water‐energy nexus, water resources management, Gaza Strip, UN sustainable development goals

## Abstract

The acute water and electricity shortages in Gaza necessitate comprehensive solutions that recognize the interconnected nature of these vital resources. This article presents pragmatic solutions to align supply with fundamental needs in both domains, offering viable pathways for achieving strategic water‐energy security in Gaza. Baseline data reveals a deficit in the current water supply, falling below the international minimum of 100 L per capita per day, while the reported 137–189 MW per day electricity supply significantly lags behind the estimated 390 MW per day peak demand. To meet projected 2024 residential, commercial, and industrial demands, this study proposes actionable measures including expanding wastewater treatment to enable over 150 MCM per year tertiary effluents for agricultural reuse and adopting energy‐efficient forward osmosis‐reverse osmosis and osmotically assisted reverse osmosis desalination methods to increase potable water supply to 150 MCM per year. Electricity supply strategies include scaling renewable capacity towards 110 MW per day, exploring regional cooperation to unlock over 360 MW of power per day, and potentially recovering up to 60 MW per day through system efficiencies. These recommendations aim to prevent exacerbated scarcity and alleviate hardships in Gaza.

## Graphical abstract

This article introduces forward‐looking solutions for Gaza's water and energy future. Following a data‐diven systems approach, the study analyzes both current and anticipated water and energy requirements, while assessing the feasibility of diverse supply alternatives. The insights significantly contribute to the understanding of the water‐energy nexus for policymakers, researchers, and practitioners engaged in the domains of enery and water resource management.

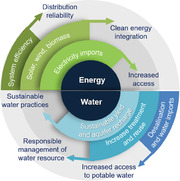

## 1 Introduction

Water and energy are intricately connected, with water extraction, distribution, and treatment requiring significant energy inputs. Conversely, energy production is heavily reliant on adequate water availability.^[^ ^1^, ^2^ ^]^ This interdependent relationship termed the “water‐energy nexus,” presents pressing challenges for effective planning. Failing to adopt a clear and strategic approach only exacerbates existing challenges, impeding the achievement of sustainable and resilient water and energy systems.

Addressing the water‐energy nexus remains a global priority for sustainable development. While countries like the United States, China, Spain, and Australia have devised comprehensive policies to confront this issue, the Gaza Strip faces unique and complex challenges characterized by limited energy resources and the overexploitation of renewable water resources.^[^ ^3^, ^4^ ^]^ With a land area of 365 square kilometers and a dense population exceeding two million residents, the Gaza Strip heavily depends on water and energy imports from neighboring countries and international support to develop these sectors. However, the effectiveness of proposed donor solutions is often impeded by a lack of precise understanding of how these solutions interact with the specific conditions prevalent in Gaza.

National and international research institutions have conducted assessments on the current situation in Gaza resulting in reports covering various topics, including the basic needs of residents and the implementation of advanced options.^[^ ^5^, ^6^ ^]^ However, discussions on critical issues, such as wastewater irrigation^[^ ^7^, ^8^, ^9^, ^10^, ^11^ ^]^ and renewable energy utilization in Gaza's water supply,^[^ ^12^, ^13^, ^14^, ^15^ ^]^ often overlook the crucial fact that transitioning to sustainable water and energy supply is contingent upon recognizing their interdependencies.

This article aims to provide a data‐driven analysis of Gaza's current and projected water and energy requirements, while evaluating the feasibility of various supply alternatives. The study follows a methodological systems framework comprising four key steps:

1. Data Collection: Compilation of comprehensive baseline data on current water and energy demand, supply, and usage patterns.
2. Gap Assessment: Identification of supply–demand gaps in the water and energy sectors.
3. Data Evaluation: Evaluation of potential options based on their technical and economic feasibility.
4. Recommendations: Proffering evidence‐based recommendations for implementing feasible and sustainable water and energy solutions in the Gaza Strip.

By following this methodological framework, this article aims to contribute to a better understanding of the water and energy challenges in the Gaza Strip, providing valuable insights for sustainable solutions that can effectively address the specific needs of this area. The article underscores the need for a paradigm shift in addressing the energy‐water nexus, emphasizing the importance of holistic planning and collaboration among relevant stakeholders to navigate the intricate dynamics of water and energy supply for a sustainable and resilient future in the Gaza Strip.

The findings presented aim to meet the basic needs stipulated by the World Health Organization (WHO) and better align with the “Green Deal” policy of the European Commission. This study is also directly linked to two of the United Nations' Sustainable Development Goals: Goal 6, which centers on Clean Water and Sanitation, and Goal 7, aimed at achieving Affordable and Clean Energy. These goals recognize the critical role of sustainable energy practices and efficient water management in achieving sustainable development.

## 2 Gaza's Water and Energy Challenges

### 2.1 Water Supply Challenges

Gaza's water supply heavily depends on the Coastal Aquifer, which contributes approximately 90 percent of the total water supply. The remaining water is sourced from Mekorot, Israel's national water company (5 percent), and seawater desalination plants (5 percent).^[^ ^16^ ^]^ Water extraction from the aquifer is facilitated by over 266 active municipal water wells operating daily, serving local communities and farms. Unfortunately, unsustainable extraction practices have resulted in alarming seawater intrusion into the aquifer.^[^ ^17^ ^]^ The Israeli Ministry of Environmental Protection reports an annual abstraction of approximately 245 MCM from the aquifer, whereas the sustainable yield is estimated at merely 5 MCM per year.^[^ ^7^ ^]^ This over‐pumping has led the United Nations to identify the water problem as a significant contributing factor to its prediction that Gaza could soon become uninhabitable.^[^ ^18^ ^]^

The daily water supply averages 110 Liters per capita per day (LCD). However, approximately 24 percent of this water is lost due to network leakage, resulting in an average water consumption per person in Gaza of roughly 84 LCD.^[^ ^19^ ^]^ This falls short of the WHO recommendations, which advocate for a minimum standard of 100 LCD and an average of 150 LCD to meet basic health needs.^[^ ^20^ ^]^ Furthermore, the extracted water is limited to non‐potable purposes, such as personal hygiene, laundry, domestic cleaning, and sanitation.^[^ ^21^ ^]^ Consequently, residents heavily depend on purchasing water from tankers and bottled sources for drinking purposes.^[^ ^22^ ^]^ Desalinated water from tankers serves as the primary household potable water source for up to 83 percent of the population, while bottled water is imported from neighboring countries.^[^ ^23^ ^]^

The Coastal Municipalities Water Utility (CMWU) is responsible for water management in Gaza, while Israel retains control over the water resources without direct involvement in local water management practices.^[^ ^17^ ^]^ Over the years, the international community has invested millions of dollars in supporting this sector, acknowledging the perceived shortcomings of the CMWU in developing a sustainable water supply system. However, significant efforts are still required to establish a truly sustainable system.

### 2.2 Wastewater Management Challenges

Wastewater management in the Gaza Strip grapples with substantial challenges, marked by an annual generation of approximately 80 MCM of domestic wastewater. Out of this total, about 83.5 percent is conveyed through a sewage network comprising 49 pump stations and four wastewater treatment plants (WWTP), while the rest is discharged through septic tank cesspits and open drains.^[^ ^24^ ^]^

Merely 9.4 MCM of the approximately 60 MCM wastewater collected undergoes treatment to a level suitable for irrigation. The remaining sewage either remains untreated or undergoes inadequate treatment, resulting in reported levels of biological oxygen demand (BOD) exceeding 300 mg/L.^[^ ^25^ ^]^ Insufficient treatment is primarily attributed to the lack of inadequate treatment facilities, unreliable electricity supply, and frequent power cuts that significantly impede the wastewater treatment process. Typically, raw sewage displays BOD values between 200 and 600 mg/L, whereas efficiently treated municipal sewage typically falls within the range of 10 to 20 mg/L.^[^ ^26^ ^]^ The Palestinian standard for BOD in treated wastewater is 60 mg/L.^[^ ^27^ ^]^

Discharging untreated or inadequately treated sewage into various water bodies, including the Mediterranean Sea, poses potential health and environmental hazards.^[^ ^28^ ^]^ The improper disposal of untreated wastewater also contributes to the degradation and contamination of the Coastal Aquifer. The infiltration of untreated wastewater into the ground elevates nitrate levels, resulting in severe health implications such as blue baby syndrome. Alarmingly, extensive areas in Gaza far exceed the WHO's recommended limit for nitrate levels.^[^ ^28^ ^]^

Overall, the combined impacts of over‐pumping and contamination have pushed the aquifer to the brink of collapse, posing a severe threat to its long‐term usability. Therefore, it is crucial to implement sustainable water extraction and wastewater treatment solutions to protect public health, preserved the aquifer, and safeguard the region's environmental well‐being.

### 2.3 Electricity Sources and Supply Challenges

Historically, the Gaza Strip has depended on Israel, Egypt, and a local power plant as its primary sources of electricity. On average, the Israel Electric Corporation supplies 120 MW of electricity daily through ten 22 kV medium voltage lines.^[^ ^29^ ^]^ Furthermore, until April 2017, the El Arish power plant in Egypt provided an additional 28 MW per day of electricity to the Strip via three 22 kV power lines south of the Gaza Strip, with the cost fully covered by the Arab League.^[^ ^30^ ^]^ However, the electricity supply from Egypt was disrupted due to violent incidents in the Sinai Peninsula and remains suspended due to deteriorated power lines.

The Gaza Power Plant (GPP) plays a crucial role in power generation, boasting a maximum generation capacity of 140 MW utilizing Qatari‐funded diesel fuel distillate No. 2 imported from Israel. Fuel is stored in two large tanks, each with a capacity of 10,000 m^3^, designed to sustain the GPP for 30 days without refilling.^[^ ^31^ ^]^ This requires a daily consumption of 670 m^3^ of diesel fuel for continuous operation.

Until October 2023, a limited supply of diesel fuel from Israel has been provided to the Gaza Strip to prevent a humanitarian crisis. Consequently, despite the GPP's 140 MW per day capacity, the plant only generates 23–71 MW of electrical power per day, consuming an average of 420 m^3^ of fuel daily at a cost ranging from 0.30‐0.46 US dollars per kWh, making it the highest price in the entire Middle East and North Africa (MENA) region.^[^ ^32^ ^]^ This cost includes only capacity charges and does not account for taxes. The residential tariff for electricity in Gaza varies between 12.6 to 12.8 US cents per kWh.

**Figure 1:**
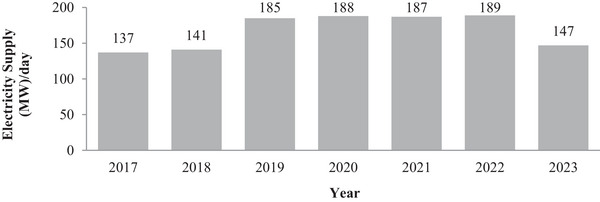
Average electricity supply 2017–2023 in MW per day, based on data from the United National Office for the Coordination of Humanitarian Affairs.

As depicted in **Figure** **1** below, the average electricity supply in Gaza ranges between 137 and 189 MW per day, resulting in a total electricity consumption of 2,100–2,900 GWh per year or 1,000–1,400 kWh per year per capita.^[^ ^33^ ^]^ By comparison, the global average electricity consumption per capita is approximately two times higher at 2,674 kWh per year. To provide further context, the average consumption per capita in 2021 was 15,294 kWh per year in Kuwait, 21,185 kWh per year in Bahrain, 13,774 kWh per year in the United Arab Emirates, 16,740 kWh per year in Qatar, 7,300 kWh per year in Oman, 7,079 kWh per year in Israel, 6,193 kWh per year in Lebanon, and 1,794 kWh per year in Jordan.^[^ ^34^ ^]^ To reach global average consumption levels, the capacity of Gaza's electricity system should be 350–390 MW per day, adding 160–210 MW per day.

To cope with the shortage of electricity supply, many residents rely on private electric generators and Solar Water Heaters (SWH). It is estimated that up to 56 percent of households have an SWH system installed on their roofs. However, approximately one‐third of these systems are reportedly non‐functional, further exacerbating the challenges faced by the population in accessing reliable and affordable electricity.^[^ ^35^ ^]^

The Gaza Electricity Distribution Company (GEDCO) is the sole power distributor responsible for billing, payment collection, and maintenance of distribution lines. However, with a sustained decline in household income and an average intermittent daily electricity supply of around 11 hours in 2023, GEDCO has encountered difficulties securing payments for electricity bills. Between 2005 and 2018, there has been a notable decline in the collection rate. While the collection rate stood at 80 percent in 2011, it decreased to approximately 65 percent by 2017 and further plummeted to 32 percent in 2018 among the company's 250,000 subscribers.^[^ ^36^ ^]^ GEDCO's low collection rate has adversely affected the maintenance of transmission lines and power transformers, resulting in distribution losses amounting to approximately 30 percent.^[^ ^29^ ^]^ Comparatively, the worldwide annual electricity transmission and distribution losses average is around 8 percent.

## 3 Gaza's Future Needs and Prospective Solutions

Addressing Gaza's essential water and energy needs requires sustainable solutions that recognize the interdependence of these vital resources. With rising demands straining already limited supplies, integrated planning around the water‐energy nexus becomes imperative. This section explores prospective solutions across both domains to align supply with interconnected system requirements. To achieve this goal, it is essential to investigate the relationship between resources, economics, and society to ensure a water‐energy nexus plan with the lowest possible carbon footprint.^[^ ^37^ ^]^ Such an approach supports progress toward more sustainable resource utilization in alignment with Gaza's development objectives.

### 3.1 Integrated Water Resources Management

Addressing Gaza's impending water crisis necessitates strategically adopting desalinated water aligned with projected population demand growth. The current water demand ranges between 73 MCM per year for a minimum of 100 LCD and 110 MCM per year for a 150 LCD supply. The projected increase in water demand through 2045 forms the basis for a comprehensive and sustainable water management plan. According to the Palestinian Central Bureau of Statistics, Gaza's current population growth rate of 2.91 percent per year is projected to double by 2045. This growth trajectory necessitates an augmented annual water supply to meet the WHO recommendation, reaching 153 MCM for a minimum of 100 LCD and 230 MCM for an average recommended 150 LCD. Notably, Gaza's population growth rate is nearly three times higher than the 1.05 percent average global rate, underscoring the urgency of tackling water supply challenges. Accounting for the declining global population growth trajectories, under a conservative 1.30 percent rate, Gaza's population in 2045 would reach 2.83 million, resulting in annual water demand of 103 MCM for a minimum of 100 LCD and 155 MCM for an average of 150 LCD.

Given the scarcity of water resources and energy supply constraints, Gaza must judiciously manage its available water resources and opt for the most suitable treatment and desalination methods. The choice of desalination technology will depend on various factors, such as energy efficiency, feed salinities, and compositions.

#### 3.1.1 Seawater Reverse Osmosis as a Desalination Method

Seawater reverse osmosis (SWRO), a widely used membrane‐based desalination method, accounts for approximately 43 percent of the world's desalination capacity.^[^ ^38^ ^]^ This process employs high‐pressure power pumps to drive seawater through semi‐permeable membranes, effectively removing dissolved solids. A typical SWRO unit requires 2.5–4 kWh/m^3^ of electric power. This power consumption is split, with half overcoming osmotic pressure, 25 percent used to overcome membrane filtration resistance, 20 percent allocated for pump and energy recovery device inefficiency, 2.5 percent lost to concentration polarization effects, and the remaining 2.5 percent accounting for friction losses in the permeate and concentrate flow lines.^[^ ^39^, ^40^ ^]^

The average worldwide leakage rate is 20 percent, marginally smaller than the reported 24 percent in the Gaza Strip. Accounting for 20 percent leaking, constructing a 150 MCM SWRO desalination plant will require 51–82 MW of electricity per day. The current electricity supply from Israel, priced at 0.16 US dollars per kWh, would have an estimated annual electricity cost of approximately $72‐115 million. However, the energy cost represents a relatively small portion of the total cost, currently at $0.5/m^3^.^[^ ^41^ ^]^ Overall, the annual operation cost for SWRO amounts to at least $90 million, equivalent to $32 per capita per year.

SWRO is most effective with feedwater derived from low‐salinity brackish water to intermediate‐salinity seawater. These resources include the Mediterranean Sea, with a salinity of 38.9 g/L, and the coastal aquifer and other underground water sources from wells, with salinity levels ranging from 1 to 7 g/L.^[^ ^42^, ^43^ ^]^ However, to maximize the utilization of limited water resources in Gaza, dewatering brine with higher salinity, such as rejected brine and some high saline wastewater, should also be considered. The feedwater salinity limits reverse osmosis's effectiveness, as typical membrane burst pressures reach a maximum salinity of 75 g/L. As the saltiness of the feedwater increases, the specific energy consumption (SEC) of SWRO also rises, especially when pre‐treatment is required to remove divalent ions and biomaterials.^[^ ^44^ ^]^ Therefore, there is an urgent need to develop alternative desalination methods capable of handling highly saline and complex feedwaters and explore strategies to optimize the utilization of rejected brine to mitigate associated costs.

#### 3.1.2 Addressing Salinity Challenges and Optimizing Water Resource Utilization

To overcome the challenges posed by higher salinity feedwater and enhance water resource utilization in Gaza, two potential options for improving reverse osmosis (RO) facilities have been identified. The first option involves hybridizing RO with another desalination process as a post‐treatment method to enhance water quality. The second option entails modifying RO to an osmotically assisted RO (OARO) process, allowing for higher water recovery without exceeding membrane burst pressures.

In contrast to reverse osmosis, forward osmosis (FO) is an emerging technology that capitalizes on the osmotic pressure gradient between draw and feed solutions to extract fresh water from seawater.^[^ ^45^ ^]^ However, while FO is energy‐efficient, the draw solution recovery process consumes a relatively large amount of energy. Direct FO is more suitable for low‐salinity feedwater, such as brackish water, and is not considered a viable desalination process for high‐salinity feedwater like seawater.^[^ ^46^ ^]^ It is also unlikely to reduce energy consumption significantly compared to RO.^[^ ^47^ ^]^ The combination of several different membrane‐based technologies, such as forward osmosis, nanofiltrations, and reverse osmosis, could further enhance water reuse and desalination performance.^[^ ^48^ ^]^

A hybridized FO‐RO system with indirect FO desalination shows excellent potential to overcome this limitation. Studies conducted by scientists from Saudi Arabia and Korea have demonstrated that FO can serve as a pre‐treatment method to reduce the salinity in feedwater.^[^ ^43^ ^]^ In this system, the feedwater acts as the draw solution, while quality‐impaired or low‐salinity wastewater serves as the feed. The FO stage is followed by a low‐pressure reverse osmosis (LPRO) stage, further reducing salinity and achieving freshwater with lower specific energy consumption.^[^ ^49^, ^50^ ^]^ This hybrid desalination system aims to utilize high‐salinity water resources and reduce the energy cost associated with conventional RO. Compared to SWRO, the FO‐RO process consumes approximately 1.3–1.5 kWh of energy per cubic meter of freshwater, roughly half of the energy consumption of SWRO. However, the FO‐RO process requires a 21 percent higher capital cost than SWRO. To meet Gaza's minimum demand for 103 MCM of fresh water per year, FO‐RO desalination would need around 15–25 MW of electricity, approximately half of the current demand, significantly alleviating the energy required to run such a system.

Another potential option for reducing energy consumption is the application of OARO to very high‐salinity water sources, such as rejected brine. This emerging technology, developed recently, is a non‐evaporative, membrane‐based process. Unlike RO, OARO allows nearly zero total dissolved solids (TDS) to permeate the membrane, and the TDS sweep on the draw side reduces the difference in osmotic pressure across the membrane. By employing multiple stages in series, OARO enables the recovery of freshwater from high‐salinity feedwater.^[^ ^51^, ^52^ ^]^ Techno‐economic analyses by Peters and Hankins demonstrate that OARO exhibits significantly lower energy consumption than conventional RO, particularly at water recovery rates above 0.55.^[^ ^53^, ^54^ ^]^ Additionally, at a 50 percent water recovery rate, OARO requires only 15 percent higher capital cost than RO and is slightly cheaper to operate than FO‐RO, although FO‐RO saves more electricity.

**Table** **1** below presents the potential enhanced desalination methods. It is essential to highlight that, in general, membrane‐based technologies require lower capital costs and specific energy consumption compared to conventional thermal or mechanical‐based desalination methods. Thermal and mechanical‐based desalination methods are not recommended due to their elevated specific energy consumption and capital cost, despite their operational adaptability across a broader salinity range. Therefore, by selecting suitable membrane desalination technologies, the water industry in Gaza can produce more affordable water and reduce energy consumption.

**Table 1:** A summary of energy and capital costs for alternative membrane desalination methods.

Desalination method	Tested salinity [g L^‐1^]	SEC at 50% recovery [kWh m^‐3^]	Capital cost (normalized to SWRO)
FO‐RO	–	1.3–1.5 ^[^ ^55^ ^]^	1.21 ^[^ ^56^ ^]^
SWRO	5‐40 ^[^ ^51^ ^]^	2.5–4.0 ^[^ ^57^ ^]^	1.00 ^[^ ^56^ ^]^
OARO	70 ^[^ ^51^ ^]^	1.8 ^[^ ^51^ ^]^	1.15 ^[^ ^53^ ^]^
MVC	35–150 ^[^ ^51^ ^]^	11–25 ^[^ ^51^ ^]^	3.55 ^[^ ^56^ ^]^
MSF	35–150 ^[^ ^51^ ^]^	13.5–25.5 ^[^ ^58^ ^]^	2.89^[^ ^56^ ^]^

^a)^ Thermal‐based desalination methods like multi‐stage flash (MSF) and mechanical‐based desalination methods, such as mechanical vapor compression (MVC) desalination, are widely utilized in the MENA region, comprising over 80 percent of the desalination capacity.^[^ ^59^ ^]^

#### 3.1.3 Proposed Water Infrastructure Upgrades

The desalination of seawater from the Mediterranean Sea can be carried out either within or outside the Gaza Strip. With its extensive expertise and five large desalination plants, Israel is a potential partner in addressing Gaza's water needs. Unlike Gaza, Israel is not constrained by energy shortages and possesses ample resources for seawater desalination to meet public demand. Notably, the Ashkelon desalination plant, located just 13 km northeast of Gaza, is globally recognized for its advanced capabilities, with an initial capacity of 120 MCM annually that expanded to 143 MCM in 2010.

**Figure 2:**
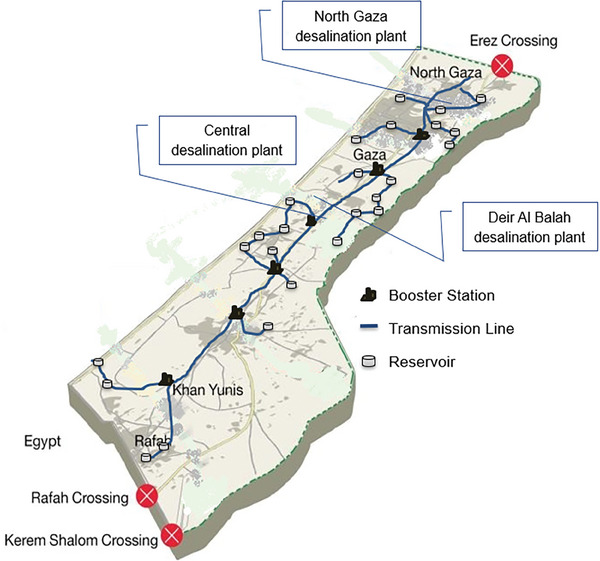
Gaza's proposed water infrastructure and desalination plants.

**Figure** **2** presents an alternative approach where water is desalinated within Gaza. This approach necessitates constructing essential infrastructure, including six booster stations, 25 reservoirs, and 85 km of pipelines, to effectively distribute desalinated water throughout Gaza, from North Gaza to Khan Younis and Rafah in the south.^[^ ^60^ ^]^

Acknowledging the crucial role of Gaza's infrastructure in future development, official Palestinian Authority documents emphasize the significance of strengthening existing plants in Deir Al Balah and North Gaza by establishing an additional large central desalination plant. The Central Desalination Plant's first line is anticipated to be operational in 2030, with the total capacity expected by 2035. For further information on existing and projected desalination capacities, refer to **Table** **2**.

**Table 2:** Existing and projected desalination capacities in Gaza through 2045 (in MCM per year).

Year	2023	2030	2035	2045
Central Desalination Plant (Planned)	–	55	55	110
Deir Al Balah Desalination Plant (Existing)	2	2	2	2
North Gaza Desalination Plant (Existing)	4	4	4	4
Total Desalination Capacity	6	61	61	116

The current desalinated water production falls short of the predicted requirement based on the WHO's recommendation of 100 LCD and a conservative population growth rate. Even with conservative population growth projections considered, it is still short by 34 MCM per year to reach the 150 MCM per year required to meet the needs of the future population. Therefore, a combination of aquifer and desalinated water sources becomes essential. As wastewater treatment capacities improve to meet required standards, allocating a blend of desalinated water with WWTP effluents recharged to the aquifer for potable uses will be feasible. This integrated approach is crucial to ensuring a sustainable and sufficient water supply for the growing population in the future.

#### 3.1.4 Optimizing Wastewater Treatment for Reuse

Wastewater is treated at four WWTPs in North Gaza, Khan Younis, Rafah, and Gaza Shikh Ejleen. Refer to **Table** **3** for existing and projected wastewater treatment capacities in Gaza. To preserve the soil's salt balance and ensure the appropriate water quality for specific crops, 71 percent of the total agricultural demand can be met by treated wastewater and the rest by aquifer water.^[^ ^61^ ^]^ Implementing these water and wastewater solutions is a time‐intensive process dependent on the availability of electricity. International collaboration and efforts are crucial to address the challenge of accessing primary energy sources.

**Table 3:** Existing and projected wastewater treatment capacities in Gaza through 2045 (in MCM per year).

Year	2023	2030	2035	2045
North Gaza WWTP (Existing)	13	22	22	22
Khan Younis WWTP (Existing)	10	10	16	16
Rafah WWTP (Existing)	4	4	4	4
Gaza WWTP (Existing)	33	33	33	33
Central Gaza WWTP (Planned)	–	–	44	60
Southern WWTP (Planned)	–	–	10	15
Total Wastewater treatment capacity	60	69	129	150

With an assumed 83.5 percent sewage collection rate and 10 percent technical losses, the available wastewater to be treated for irrigation in the Gaza Strip exceeds 60 MCM per year. As the water supply increases, it becomes imperative to enhance the capacity of existing WWTPs and commission additional facilities for sustainable outcomes. To achieve this, sewage must undergo a three‐stage treatment process to reduce organic and microbiological loads to manageable levels, allowing for the safe release or reuse of effluents. This reduction is accomplished using biomass in bioreactors, including aerated lagoons, trickling filters, constructed wetlands, membrane bioreactors (MBRs), or conventional activated sludge (CAS). Among these options, CAS is one of the oldest and most popular methods, employing freely floating flocs of aerobic bacteria to decompose dissolved organics. CAS consists of three stages: 1) mechanical treatment, 2) biological treatment, and 3) filtration and chlorination.

The primary treatment stage involves wastewater collection, pumping, screening, grit removal, and sedimentation in the primary tank sedimentation. Apart from wastewater pumping, the immediate treatment stage requires relatively low energy, with average energy demands ranging from 0.02 to 0.1 kWh/m^3^ in Canada, 0.045 to 0.14 kWh/m^3^ in Hungary, and 0.1 to 0.37 kWh/m^3^ in Australia.^[^ ^62^, ^63^, ^64^ ^]^

The secondary treatment, which includes aeration, mixing activated sludge in denitrification basins, and sludge recirculation (pumping), is the primary energy‐consuming process in wastewater treatment and discharge. The average energy demand for secondary treatment ranges from 0.46 kWh m^3^ in Australia, 0.27 kWh/m^3^ in China, 0.47 kWh/m^3^ in the United States, to 0.30–1.89 kWh/m^3^ in Japan.^[^ ^65^ ^]^ Surface aeration is the largest energy consumer. Recent studies show that introducing micro nanobubbles reduces energy consumption and improves oxygen transfer rate.^[^ ^66^, ^67^, ^68^ ^]^

For tertiary treatment, such as chlorination or sand filtration, an additional 0.2 kWh m^−3^ is required for filtration alone, while the entire coagulation–flocculation–sedimentation–filtration process may require up to 0.5 kWh m^3^. Chlorination has a relatively low energy demand, ranging from 2 × 10^−5^ to 2 × 10^−3^ kWh/m^3^.^[^ ^69^ ^]^ Assuming an average energy consumption of 0.8 kWh/m^3^ for advanced wastewater treatment to meet the required quality level for unrestricted irrigation, a total capacity demand of 5.5 MW would be necessary.

The 60 MCM treated wastewater would primarily be used for fruit tree irrigation. The daily water requirements per tree vary according to the season, tree type, and evaporation rate. Recommended daily rates range from 16 to 62 L per tree, averaging 35 L/day/tree or 12 m^3^/year/tree.^[^ ^70^ ^]^ For the chief crop, citrus fruit, with a planting density of 6×6 m per tree or 270 trees per hectare (10,000 m^2^), Gaza's total area of 365 km^2^ would be sufficient to meet the irrigation needs of approximately 4.9 million trees if 50 percent of the entire area is dedicated to citrus tree cultivation. If the effluents are used for crops with higher water demands, such as wheat or potatoes, they can be blended with water from the aquifer while still meeting water quality requirements.^[^ ^71^ ^]^

### 3.2 Addressing Gaza's Growing Energy Demand

**Figure 3:**
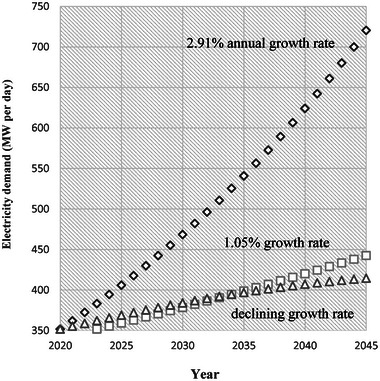
Predicted electricity demand through 2045 under different population growth rates (in MW per day).

The current electricity supply of 1,000–1,400 kWh per year per capita is approximately half the global average of 2,674 kWh. Extrapolating the current supply to the global average, considering the reported 2.91 percent yearly population growth rate, indicates a projected total demand of 720 MW per day by 2045. With a more conservative 1.30 percent annual population growth rate, the energy demand is estimated to be 500 MW per day, aligning closely with the average electricity supply‐demand values outlined by the United Nations Office for the Coordination of Humanitarian Affairs based on data sourced from GEDCO, the official body in charge of electricity supply in the Gaza Strip.^[^ ^72^ ^]^ This projection includes around 150 MW per day for water, wastewater, and desalination facilities, based on operational requirements outlined in Section 3.1 and Tables 2 and 3 above. An additional 100 MW per day will be required to meet residential demand, based on the reported average annual electricity consumption of 10 000 kWh per household for GEDCO's 250,000 subscribers. The remaining demand, ranging between 250 MW and 470 MW per day, as illustrated in **Figure** **3**, is uncertain and depends on the future needs of the commercial and industrial sectors. These demand forecasts are contingent on extrapolating the current demand with expected population growth, indicating an imminent surge that is two to three times the pre‐ October 7, 2023, electricity supply to the Gaza Stip.

To address Gaza's electricity deficit, immediate‐term options should be explored. One option involves renewing and expanding the electricity supply from Egypt, with agreements to restore the 28 MW supply through the Canal Electricity Distribution Company from the Al‐Arish Power station in the Sinai Peninsula. This option faces challenges due to inadequate infrastructure in Gaza and the need for substantial transmission line expansion.

A compelling near‐term option with significant potential is the rapid deployment of solar energy systems. Gaza's abundant sunlight makes solar power an ideal and readily available renewable energy source. By installing solar generators on rooftops and establishing dedicated solar farms, Gaza could quickly augment its electricity generation capacity by up to 100 MW.

Rooftop solar installations offer a pragmatic solution for households, businesses, and institutions to achieve self‐sufficiency in electricity generation. Furthermore, developing dedicated solar farms will enhance overall energy resilience. Local authorities and international organizations have recently explored this option, supported by financial incentives, grants, and technical support for solar projects. An exemplary illustration is the $11 million World Bank project initiated in 2018, which provided loans to households and businesses and grants for essential public services such as hospitals, facilitating the installation of rooftop solar panels. Another initiative, a $2 million United Nations Development Programme project, successfully installed solar systems in four Gaza hospitals.

The cumulative installations of rooftop solar systems across critical infrastructure, businesses, and domestic settings have propelled Gaza to boast the highest density of rooftop solar systems globally. An analysis by the Center for Strategic and International Studies analysis, utilizing satellite imagery from May 2022, identified at least 655 rooftop solar systems in a one‐square‐mile sample area of Gaza City, with an estimated 12,445 rooftop solar systems.^[^ ^73^ ^]^ This remarkable progress underscores the region's potential for becoming a trailblazer in sustainable energy solutions.

In the short term, Gaza can also enhance its energy supply by constructing a 161 kV high voltage line from Israel, providing an additional 100 MW of electricity at lower tariffs, thus boosting the commercial viability of the energy sector. Discussions with Israeli authorities and international donors have commenced, but upgrading the Gaza North Substation is essential to expediting the project's implementation. Challenges include repairing damaged infrastructure and resolving uncertainties surrounding additional power costs.

Addressing non‐technical losses, such as revenue collection, can be achieved through implementing pre‐paid meters for non‐paying subscribers. In conjunction, audited accounts for revenue collection can ensure proper payment to suppliers and foster sector development. Upgrading the Supervisory Control and Data Acquisition system can improve electricity distribution efficiency and reduce losses. Easing GEDCO's financial constraints will facilitate network upgrades, allowing the potential recovery of up to 60 MW of power through system efficiencies.

In the medium term, upgrading the GPP production capacity from 140 to 300 MW by developing a natural gas pipeline from Israel presents a cost‐effective solution.^[^ ^31^ ^]^ This solution comes at an estimated 12.8 cents per kWh, significantly lower than 30 cents per kWh. However, challenges such as proper infrastructure development, uncertainties regarding the pipeline route, and concerns about the potential misuse of methane for creating explosives make this option more suitable for future exploration rather than an immediate short‐term solution.

Biomass energy also holds promise as a medium‐term option, offering up to 10 MW per day of power. Despite initial cost concerns, biomass can utilize the significant residue from the region's agricultural sector, providing a reliable and sustainable energy source.

For long‐term planning, constructing a new gas‐fired power plant in southern Gaza and implementing energy efficiency measures should be considered. Upgrading infrastructure across the Strip, ensuring major docking points with Israel and Egypt, and high throughput capacity will be essential. These infrastructure upgrades are integral to paving the way for a more sustainable and resilient energy future for Gaza.

#### 3.2.1 Exploring Solar Energy Integration

As discussed in Section 3.2 above, integrating renewable energy sources presents a promising short‐term solution for addressing Gaza's energy supply challenges. This approach offers numerous potential benefits, such as increased energy independence and reduced environmental impact from reduced reliance on fossil fuels. A detailed examination of the costs associated with alternative renewable energy technologies in the Gaza Strip is provided in **Table** **4** below. Among the renewable options, photovoltaic (PV) systems stand out due to their advantages, including low capital costs, operating and maintenance expenses, and the absence of variable operating and maintenance expenditures.

**Table 4:** Cost of alternative renewable energy technologies.

	Rooftop solar^b)^	Utility scale PV	CSP^a)^	Wind	Biomass
Capital Costs ($ per KW)	1,500	1,000	3,000	1,290	3,750
Fixed O&M ($ per KW‐Yr)	10	8	40	49	107
Variable O&M ($ per MWh)	–	–	4	–	5

^a)^ CSP, Concentrated Solar Power, used in beam solar radiation;

^b)^ According to the U.S. National Renewable Energy Laboratory annual technology baseline.

Blessed with approximately 3,000 sun hours annually, the Gaza Strip is conducive to solar energy generation. The average solar radiation ranges from 5.4 kWh/m^2^‐day on horizontal surfaces to 6.21 kWh/m^2^‐day on south‐facing tilted rough surfaces.^[^ ^31^ ^]^ These values are comparable to other regions globally with large‐scale photovoltaic installations. For instance, Madrid has an average of 4.88 kWh/m^2^‐day, Denver, Colorado, has 5.93 kWh/m^2^‐day, Sydney, Australia has 4.64 kWh/m^2^‐day, and Mexico City has 5.0 kWh/m^2^‐day.^[^ ^74^, ^75^, ^76^ ^]^ PV systems maintain power generation in all weather conditions, delivering up to 80 percent of their maximum potential output on partly cloudy days and 25 percent on overcast days.

**Figure 4:**
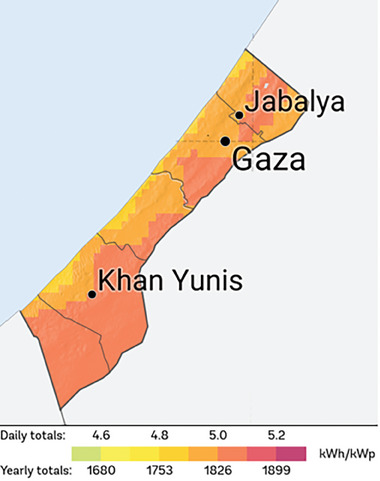
Photovoltaic power potential in the Gaza Strip.

**Figure** **4** illustrates the photovoltaic power potential derived from the Global Solar Atlas,^[^ ^77^ ^]^ a reliable source for evaluating solar resources to support solar power projects. Based on solar irradiation levels, the Gaza Strip can be categorized into two areas: a coastal region with a lower PV output of 1,753 kWh/kWp and an inner area with a higher yearly yield of 1,826 kWh/kWp. The inner area consistently maintains significant PV potential throughout the Strip.

**Figure 5:**
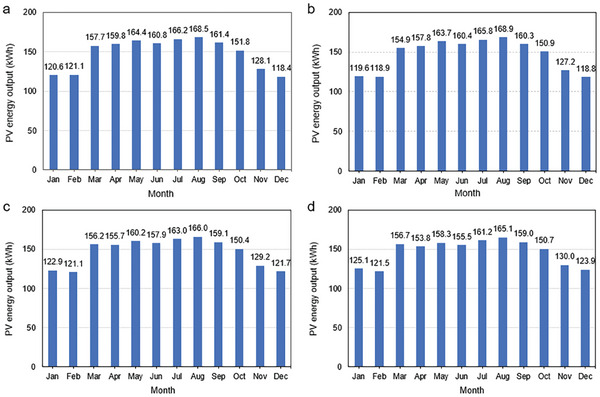
Monthly energy output of fixed‐angle PV systems in different Gaza regions: a) North Gaza, b) Gaza City, c) Deir Al Balah, and d) Rafah.

**Figure** **5** displays the energy output of fixed‐angle PV systems across different regions in Gaza. The calculations were performed using the Photovoltaic geographical information system of the EU,^[^ ^78^ ^]^ assuming a $10,000 investment for a single house, a 3 percent interest rate, and a 20‐year lifetime.

Detached PV systems emerge as an effective solution for meeting the electricity needs of residential and public buildings. With Gaza's total estimated roof surface being 21.78 km^2^, a mere 3.9 km^2^ of rooftop area is required, representing less than 10 percent of the available space.^[^ ^73^ ^]^ Refer to **Table** **5** for a detailed breakdown of the total rooftop area across five regions: North Gaza, Gaza, Deir Al Balah, Khan Younis, and Rafah. Calculations are based on an average available rooftop area of 192 m^2^ per housing unit or building, 10,000 m^2^ per school, and 100,000 m^2^ per hospital or university.^[^ ^79^ ^]^

**Table 5:** Available rooftop area in Gaza, calculated based on data provided in.^[^ ^31^ ^]^

	Building	Hospitals	Schools	University	Rooftop, km^2^
North Gaza	10,919	5	123	0	3.83
Gaza	21,750	14	267	6	8.85
Deir Al Balah	7,258	2	100	1	2.69
Khan Younis	9,470	6	140	2	4.02
Rafah	6,019	3	84	1	2.40
Gaza Strip (total)	55,416	30	714	10	21.78

The installation process for detached PV systems is straightforward, requiring minimal maintenance and a 20‐year lifespan. With no moving parts, visual inspections and battery servicing make maintenance feasible. However, the estimated $10,000 installation cost for a typical household with a maximum power of 10 kW and a 60 m^2^ horizontal roof surface represents a substantial capital investment. Despite this, detached PV systems find applications in various sectors, including water supply in villages, irrigation, and lifting water from a distribution pump to building roofs. However, specific challenges need to be addressed for the widespread adoption of decentralized PV installations. These challenges include limited local assembly and manufacturing capacity, distribution issues, installation and maintenance constraints, low consumer awareness, lack of appropriate financing schemes, and prohibitive initial capital costs, all of which raise concerns regarding the viability of these systems.

Centralized, grid‐connected solar systems offer the potential to reduce electric utility costs and generate income. Centralized electricity generation through PV can be achieved using existing rough surface areas or dedicated solar farms. **Table** **6** compares two system design options with 250, 450, and 650 MW DC system capacities at standard test conditions. The calculations were conducted using the System Advisory Model software developed by the U.S. National Renewable Energy Laboratory.^[^ ^80^ ^]^

**Table 6:** Metrics of different PV installations.

	250 MW		450 MW		650 MW	
	TAT	FRM	TAT	FRM	TAT	FRM
Annual energy GWh	443	624	798	1,123	1,152	8.02
Energy yield, kWh per kW	1,773	2,497	1,773	2,497	1,773	2,497
LCOE, cents per kWh	5.58	3.96	5.58	3.96	5.58	3.96
Net capital cost, million $	268	268	482	482	696	696
Equity, million $	366	366	660	660	953	953
Size of debt, million $	‐98	‐98	‐178	‐178	‐257	‐257

Data for a typical meteorological year was specifically collected for Gaza City. The modeling utilized a standard crystalline silicon module with a glass cover featuring an approximate nominal efficiency of 17 percent, a temperature coefficient of power of ‐0.47 percent/°C, and a fill factor of 77.1 percent (considering self‐shading). Two array types were employed: fixed roof mount (FRM) and 2‐axis tracking (TAT). FRM represents typical residential installations with modules attached to the roof surface with limited airflow between the module back and the roof surface. Conversely, TAT rotates from east to west throughout the day to track the sun's movement across the sky and follow the sun's seasonal trajectory from north to south.

Larger installations increase costs, ranging from $98 million for a 250 MW installation to $257 million for a 650 MW installation. The overall installation cost was correlated with the net capital cost, amounting to $268 million for the 250 MW installation and $696 million for the 650 MW installation. However, the installation size did not affect the levelized cost of electricity, which depended solely on the array type. The cost of electricity for FRM was 5.58 cents per kWh, whereas TAT was 3.96 cents per kWh, making it four to eleven times cheaper than the current electricity generated by the GPP, which costs 0.29–0.46 US $ per kWh.^[^ ^32^ ^]^

The establishment of solar farms and electricity distribution through GEDCO would require a significant area of 3.9 km^2^, nearly one percent of the 365 km^2^ Strip's territory, to generate an estimated 650 MW of electricity. Such an area does not exist within the Gaza Strip, requiring the solar farm to possiably be established in the Negev Desert in Israel or the Sinai Peninsula in Egypt. This option demands a more explicit financing mechanism and is subject to potential legal, regulatory, and grid‐related challenges. Moreover, many rural villages in Gaza are not connected to the central grid, and the overall grid quality has deteriorated due to a lack of maintenance. A rooftop installation would be more straightforward and economically viable than connecting to the central grid.

#### 3.2.2 Exploring the Prospect of Biomass Energy

Among the range of renewable energy possibilities, biomass energy also presents promising prospects in Gaza. Biomass is associated with the highest capital costs and operational expenses among the available energy options. Agriculture plays a pivotal role in Gaza's economy, contributing significantly to gross domestic product through commodity exports and employing a large workforce. The agricultural sector generates substantial residue that can be harnessed for various purposes, such as cooking, heating, or electricity production through biomass gasification. Fruit tree lignocellulose can be used to produce biodiesel, with the biomass‐to‐biodiesel conversion process yielding approximately 200 L of synthetic diesel per dry ton of residue.^[^ ^81^ ^]^ This amount is equivalent to generating 22,800 tons of diesel from total agricultural residue, potentially fulfilling up to 10 percent of the Strip's diesel supply requirement.

#### 3.2.3 Exploring the Feasibility of Wind Energy Integration

The final renewable energy source explored is wind power. To assess the feasibility and efficiency of harnessing wind energy in Gaza, it is imperative to analyze key metrics such as wind speed and wind power density. Wind speed indicates the horizontal movement of air, directly influencing the energy output of turbines. Wind power density quantifies available power, offering insights into the energy potential of a given area. Consistently high values in both metrics are imperative for the feasibility of wind energy projects.

**Figure 6:**
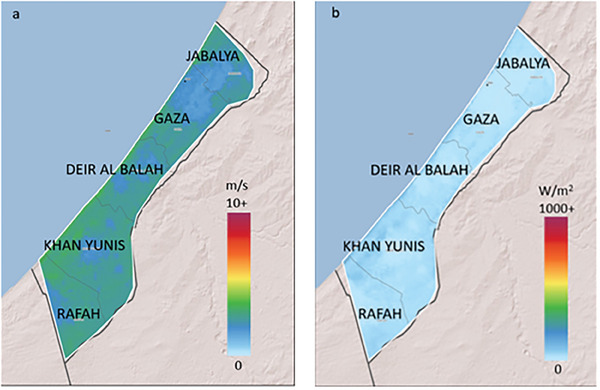
a) Mean wind speed and b) mean power density in Gaza.

Generated from the Global Wind Atlas,^[^ ^82^ ^]^ **Figure** **6** illustrates Gaza's wind speeds ranging from 2.53 to 3.43 m/s at 10 m height, with corresponding wind power density of 37.22–79.04 W m^−2^. At 50 m above ground surface, wind speeds marginally increase to 4.54–4.98 m/s, and wind power density spans 120.72–157.31 W/m^2^. With reference to NREL standards, Gaza wind potential at both 10 and 50 m above ground aligns with “poor” renewable resource classification, considering wind speed and power density parameters. While higher elevations show minor improvements, the overall values remain at the lower threshold of viability for wind energy generation.

The analysis collectively suggests suboptimal conditions for wind generation, aligning with NREL's lowest viability category. However, ongoing innovations and further exploration of elevated installations may unveil pathways to harness Gaza's latent wind resource as a sustainable energy supply. Vigilant monitoring of technological advancements, combined with updated field data, holds the potential to determine if shifting dynamics allow for a reassessment of wind energy's feasibility in the region.

## 4 Conclusion

The water‐energy nexus in Gaza poses a multifaceted and pressing challenge, requiring strategic convergence of sustainable water management and clean energy integration to secure a harmonized, sustainable, and resilient future for its residents.

Employing energy‐efficient desalination methods provides a substantial opportunity to address the burgeoning water demand. Hybridized forward osmosis‐reverse osmosis systems emerge as a promising solution, leveraging high‐salinity water resources and reducing energy costs associated with conventional reverse osmosis. Another viable option for reducing energy consumption involves the application of osmotically assisted reverse osmosis, enabling the recovery of freshwater from high‐salinity feedwater at significantly lower energy consumption. Through technology optimization and the transition to low‐energy membrane‐based desalination, Gaza can increase its potable water supply to 150 MCM per year while curbing energy consumption in desalination processes.

Expanded wastewater treatment offers an additional avenue, enabling over 150 MCM per year for agricultural demands. This strategic approach involves a judicious blend of established methods, such as conventional activated sludge, and cutting‐edge technologies, like membrane bioreactors and micro nanobubbles, aligning with international best practices. Advanced treatment processes reduce reliance on freshwater sources and promote a circular and sustainable water economy.

Addressing current and future energy needs requires the integration of renewable energy sources to provide over 110 MW of power per day, alongside increased electricity imports from Israel and Egypt. Through rooftop installations and dedicated solar farms, the pragmatic deployment of solar energy holds promise for augmenting the electricity supply by 100 MW per day in the short and medium term. The strategic use of biomass energy, utilizing agricultural residue, offers an additional 10 MW per day for diversifying the energy mix. Near‐term measures, such as renewing and expanding the electricity supply from Egypt and expediting the implementation of a 161 kV high voltage line from Israel, can provide 100 MW per day. Augmenting the local power plant's production capacity from 140 MW to 300 MW per day through a natural gas pipeline from Israel and implementing stringent energy efficiency measures are additional viable interventions. These endeavors necessitate strategic infrastructure upgrades and regional collaboration to ensure Gaza's sustainable and resilient energy future.

While this paper offers valuable insights, it is important to underscore the importance of holistic planning and collaboration among relevant stakeholders to navigate the intricate dynamics of water and energy supply. Equally important is the integration of individual projects within a comprehensive national plan to attract private investment. The study focused on data‐based evidence and quantitative analysis, deliberately avoiding discussions of the political and security situation in Gaza. The intentional focus aims to provide information supporting informed decision‐making in addressing the challenges and opportunities associated with water and energy dynamics in the Gaza Strip.

## Conflict of Interest

The authors declare no conflict of interest.

## Data Availability

The data that support the findings of this study are available from the corresponding author upon reasonable request.
